# Evolutionary Perspective in Child Growth

**DOI:** 10.5041/RMMJ.10057

**Published:** 2011-07-31

**Authors:** Ze’ev Hochberg

**Affiliations:** Meyer Children’s Hospital, Rambam Medical Center, and Rappaport Family Faculty of Medicine and Research Institute, Technion – Israel Institute of Technology, Haifa, Israel

**Keywords:** Child growth, evolution, plasticity, life-history

## Abstract

Hereditary, environmental, and stochastic factors determine a child’s growth in his unique environment, but their relative contribution to the phenotypic outcome and the extent of stochastic programming that is required to alter human phenotypes is not known because few data are available. This is an attempt to use evolutionary life-history theory in understanding child growth in a broad evolutionary perspective, using the data and theory of evolutionary predictive adaptive growth-related strategies. Transitions from one life-history phase to the next have inherent adaptive plasticity in their timing. Humans evolved to withstand energy crises by decreasing their body size, and evolutionary short-term adaptations to energy crises utilize a plasticity that modifies the timing of transition from infancy into childhood, culminating in short stature in times of energy crisis. Transition to juvenility is part of a strategy of conversion from a period of total dependence on the family and tribe for provision and security to self-supply, and a degree of adaptive plasticity is provided and determines body composition. Transition to adolescence entails plasticity in adapting to energy resources, other environmental cues, and the social needs of the maturing adolescent to determine life-span and the period of fecundity and fertility. Fundamental questions are raised by a life-history approach to the unique growth pattern of each child in his given genetic background and current environment.

When comparing the growth of a human child with that of a cat, or even that of great apes, the pattern difference is obvious (Figure 1). The concave pattern (initially slow and accelerating) of non-human mammals during their early growth is strikingly different from the convex (initially fast) human growth, followed by a linear growth pattern. This is produced by the uniquely decelerating human infantile growth and the quasi-linear childhood stage.

**Figure 1:**
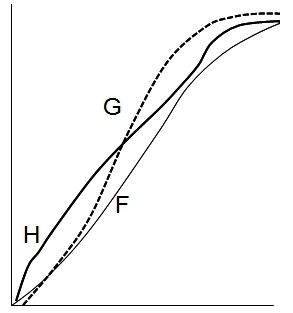
The unique human growth pattern. Human’s height pattern (H) is compared to cat’s weight (F – feline, thin line) and gorilla’s weight (G – dotted line). The concave pattern of accelerating infantile growth of both cats and apes is in contrast to the convex pattern of decelerating infantile growth in humans. Note also the unique human brisk growth acceleration during human adolescence.

Whereas several human growth processes are identical to those found in the animals’ kingdom, hominids’ life-history is markedly different (Figure 2). Humans are born immature, helpless and defenseless, have a relatively short period of infancy, and are the only species that has a childhood – a biologically and behaviorally distinct and growth-stable interval between infancy and the juvenile period that follows. We are also the only species to have true adolescence as a period devoted to puberty and accelerated growth.

**Figure 2:**
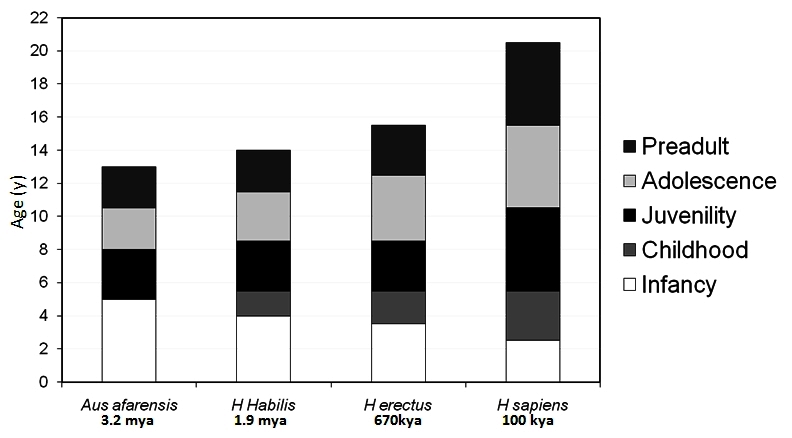
The evolution of hominid life-history during the first 20 years of life. The existence time is given below (kya, thousands years ago; mya, millions years ago) and the longevity above. During the evolution of the hominids, childhood and adolescence have been added as new life-history stages as compared with apes and the presumably early hominid *Australopithecus afarensis*. The chimpanzee serves as a living representative of the assumed *Australopithecus afarensis* life-history. As childhood emerged and prolonged, infancy was gradually cut shorter, and the latest-introduced adolescence came at the expense of a shorter juvenility.

The transition from one life-history stage to the next requires a switch mechanism for the onset of the latter, and these switches speak the language of hormones, as shown in Figure 3 for sex hormones. Note the rise in sex hormones in early infancy – the so-called “mini-puberty”, to be followed by childhood that is characterized by quiescence of sex hormones. During the following juvenile stage adrenal androgens appear, and adolescence is associated with an increase in gonadotropins and gonadal sex hormones, manifesting as puberty. It is hormones that transduce environmental information to regulate transitions between life-history stages.^1^ Indeed, most hormones have pleiotropic and often antagonistic effects on a variety of behavioral, physiological, and morphological traits. Multiple hormone mechanisms have evolved to activate behavioral and physiological traits at the right time and in the correct context. When traits are expressed throughout life-history, hormones may also potentially de-activate them for short periods.

**Figure 3:**
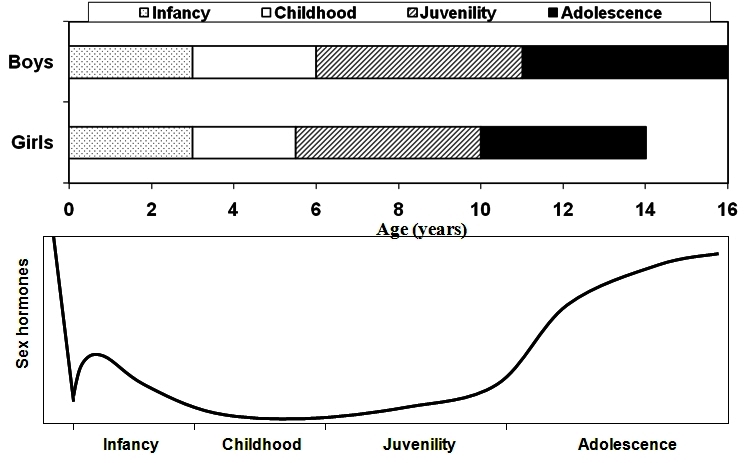
Changing sex hormone levels during the first 20 years of human life-history. Life-history stages of boys and girls (upper panel) may be defined by sex hormone levels (lower panel). Note the rise in sex hormones in early infancy – the so-called “mini-puberty”. Childhood is characterized by quiescence of sex hormones, followed by a juvenile increase of adrenal androgens and adolescent increase in gonadotropin and gonadal sex hormones, manifesting as puberty.

Life-history evolutionary theory seeks to understand the factors that produce variations in organisms’ life stages that are found both among and within species. In the case of hominids, life-history theory is a holistic attempt to integrate all fields of biology at the molecular, cellular, and organism levels with social sciences, anthropology, evolutionary biology, and psychology – and more recently also clinical medicine. Using a life-history approach, we consider the ways that evolution has worked upon these life stages to produce the adaptations of a society way of life to its environment. This is best understood in the context of biological rationale and cultural expressions as a solution to an ecological problem posed by the environment, and subject to constraints intrinsic to humans.

Two essential assumptions of life-history theory are that 1) there are set measures of fitness (a combination of survivorship and reproductive rate: individuals with higher fitness propagate more genes to future generations) that are maximized by natural selection, and 2) these are often associated with trade-offs among traits that limit the adaptive potential of a population concurrently or at a later time. Thus, species that maximize life-history traits, such as fertility, typically cannot simultaneously maximize survival. In the growth domain, species that maximize offspring size cannot maximize offspring number at the same time. Survival is affected among other by investment in immune function and adipose deposits, whereas body size is achieved among other by the function of the growth hormone – insulin-like growth factor 1 axis. The latter stimulates growth – size – while at the same time depleting adipose depots and suppressing immune function – survival. Indeed, transgenic animal studies have shown that excess growth hormone shortens the life-span, and growth hormone deficiency prolongs it. Thus, a trade-off exists between body size and survival.

This review is an attempt to use evolutionary life-history theory in the understanding of child growth in a broad evolutionary perspective with a special emphasis on the clinical aspects of this theory.

## CHILD GROWTH AND THE THEORY OF LIFE-HISTORY

Life-history has been defined as the strategic allocation of an organism’s energy toward growth, maintenance, reproduction, raising offspring to independence, and avoiding death.^2^ For a mammal, it is, among others, the strategy of when to be born, when to be weaned, when to be independent for self-protection and provision, when to accelerate or decelerate growth and when stop growing, when and how often to reproduce, and when to die in the best way as to increase fitness.^3^ In terms of child growth, life-history models assume that parents make investment decisions that maximize reproductive fitness at the cost of constraints, with a basic rule that energy, effort, and resources that are invested in the assignment growth cannot be invested in producing more offspring.^4^ As a consequence, a fundamental trade-off is between the size and the fertility rate, with larger mammals having fewer offspring.^5^

Relative to other mammals, primates, with humans at its extreme, are slow-growing, late-reproducing, long-lived, and large-brained.^6^ This package is essential to mature to function in 1) the vast variation of environments, and 2) the complexity of human society. Other distinctive characteristics of human life-histories include: 1) A period of extreme immaturity after birth which renders the newborn helpless and defenseless without his mother nearby; 2) An extended period of dependence of the young beyond breast-feeding age, while the mother becomes pregnant again, resulting in families with multiple dependent children of different ages; 3) Multi-generational resource flows and support of reproduction by older post-reproductive individuals; 4) Male support of reproduction through the provisioning of females and their offspring; and 5) The brain size and its attendant functional abilities as a central evolutionary target among humans.^7^

## LIFE-HISTORY STAGES

During the evolution of the hominids, childhood and adolescence have been added as new life-history stages as compared with apes and our ancestors (Figure 3). Genetic and anatomical evidence suggests that *Homo sapiens* arose in Africa between 200 and 100 kya, and recent evidence suggests that complex cognition may have appeared around 164–75 kya.^8^ Thus, *Homo sapiens* have five prolonged and pronounced postnatal pre-adult life-history stages: infancy, which extends for 30–36 months and ends (in natural fertility societies) with weaning from breast-feeding, when the mother becomes pregnant again; childhood, which extends for an additional 3–5 years, culminating in a degree of independence for protection and food provision; a juvenile stage, which lasts for 3–4 years, and concludes with the readiness for a sexual maturation process;^9^,^10^ and adolescence, which lasts for 3–5 years; culminating in fecundity (potential reproduction) at an average age of 17 for boys and 15 for girls. Yet, the 17-year-old boy and the 15-year-old girl are not fertile in terms of actual reproduction. It will be many more years before they pursue reproduction in modern industrial societies. But even among pre-industrial societies, it takes an average of 4 years for fecundity to be realized as fertility, later in boys than in girls in most traditional societies,^11^ and I label this new life-history phase “pre-adulthood”.

By reconstruction of indirect evidence, and without certitude, it has been suggested that as late as 3–4 mya the early hominid *Australopithecus afarensis*, best known to include “Lucy” from Hadar in Ethiopia, had merely three postnatal, pre-adult life-history stages: infancy, juvenility, and pre-adulthood. It is supposed that these hominids had a long infancy of about 5–6 years but a comparatively short life-span, and first molar teeth erupted in mid-infancy.

With a marked growth of brain size, 1.9 mya *Homo habilis* had to be born less mature to fit into a narrow bipedal maternal pelvis – the so-called obstetrical dilemma. Walking as much as 20 km a day in the open savanna, he also evolved to have longer legs than his tree-dwelling predecessors did. He had a shorter period of infancy and developed a new strategic life-history stage: childhood, with its onset defined by weaning from breast-feeding, slowing and stabilization of growth velocity, and dependence on older people other than the mother for food provision and protection.^2^,^12^,^13^

While the brain size steadily grew over evolutionary ages (3-fold as compared to other apes), newborns had to be born less mature to fit through the birth canal. Infancy grew shorter and childhood had been thriving and extending, with the first molar progressively deferred in *Homo sapiens* into the end of childhood and the transition to juvenility. As reconstructions suggest, *Homo sapiens sapiens* (from 100 kya) also introduced adolescence as a distinct life-history stage, with the typical pubertal growth spurt and rapid sexual puberty, deferring the assumption of adulthood in a species that lives longer than his predecessors. The entire assemblage has been extremely successful, and the resulting over-population of humans is, for better or for worse, the consequence of that winning strategy.

## TRANSITIONS BETWEEN LIFE-HISTORY STAGES

The developmental life of an animal may be profoundly affected by events during a short critical interval of its life – the so-called critical periods of development.^14^ Exposure to deleterious environmental compounds or child abuse during such critical periods can induce specific developmental alterations, e.g. disease susceptibility, to the offspring, and such disease susceptibility can be transmitted to subsequent generations. Transitions between life-history stages are such critical periods of development.

Multiple hormone mechanisms have evolved to activate both physiological and behavioral traits at the right time and in the correct context, as the child transits from infancy to childhood, then to juvenility, and later to adolescence and pre-adulthood. With detailed investigation of organisms’ life-history in their natural habitat, one can determine the potential ecological costs underlying hormone–physiology–behavior interactions at stage transitions that, in turn, shed light on their evolution.^15^

**Figure 4:**
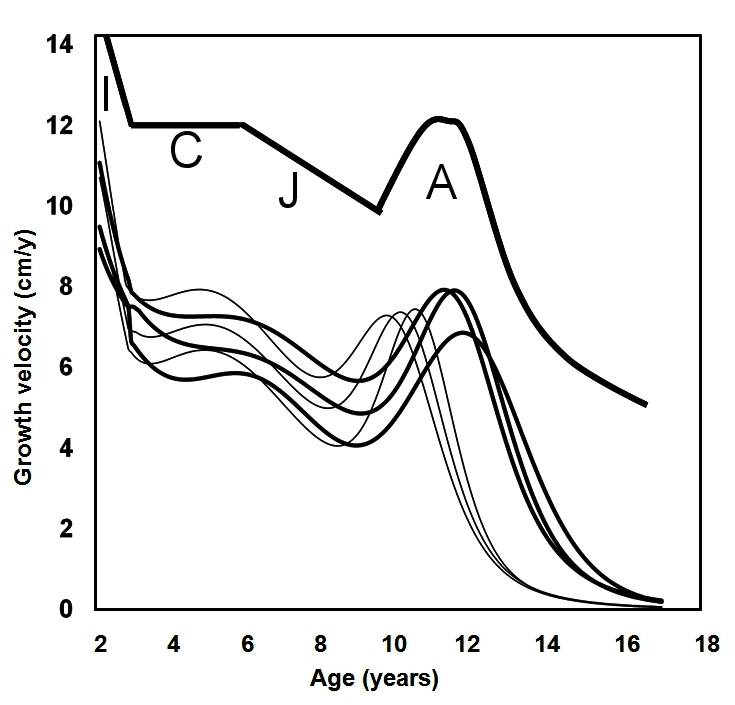
The first derivative of human height growth velocity charts. The 3rd, 50th, and 97th percentile of first derivatives for both boys (solid lines) and girls (thin lines) were calculated from the US CDC 2000 data (http://www.cdc.gov/growthcharts). The upper part indicates the four pre-adult life-history stages: infancy (I), with its decelerating growth velocity, the stable growth of childhood (C), the decelerating growth of juvenility (J), and the up and down of growth velocity during adolescence (A).

Analysis of growth through derivation of observed measurements allows for plotting the growth velocity chart (Figure 4). The stages of child growth become evident, with the rapid deceleration of infantile growth, stable growth during childhood, deceleration of growth during juvenility, and acceleration of growth during adolescence, when it reaches the peak growth velocity, before deceleration to cease growing during pre-adulthood.

## DEVELOPMENTAL PLASTICITY AND ADAPTATION

A given genotype can generate multiple phenotypes (polyphenism) depending on the environmental conditions experienced by the organism during development. These different results define plasticity that includes adaptive accommodation in all aspects of the phenotype, including morphology as well as physiology and behavior. Plasticity in developmental programming has evolved in order to provide the best chances of survival and reproductive success to the organism in an ever-changing environment. The role of flexibility in facilitating evolutionary changes has been noted by the American philosopher and psychologist James Mark Baldwin (1861–1934), who called it “organic selection” or “functional selection”, also known as the Baldwin effect, emancipating it from the models inspired by “divine pre-establishment” (Spinoza, 1632–1677). Over the years this was called “regulative ability”, “phenotypic compensation”, “ontogenetic buffering”, and “evolvability”. But whatever the name, phenotypic accommodation is the adaptive plasticity in adjusting variable aspects of the phenotype, without change of the gene sequence.^16^ This definition is much in line with the modern concept of epigenetics. Phenotypic accommodation can facilitate the evolution of novel morphology by alleviating the negative effects of change and by giving a head start to adaptive evolution in a new direction. It is the result of adaptive developmental responses, so that the novel morphologies that result are not random variants, but to some degree reflect past functionality.^16^

The *Homo sapiens sapiens* has been quite unique among organisms as a single species that lives under a variety of environmental conditions unprecedented in nature, including the entire range of geographical latitudes and altitudes, as well as extremely diverse weather conditions. Whereas the above vary slowly over generations, nutritional conditions may change more rapidly, and evolution has provided for mechanisms to adapt to these extremes, while socio-cultural adjustments filled the remaining gaps when the environment changes faster than the evolutionary time-scale.

**Figure 5:**
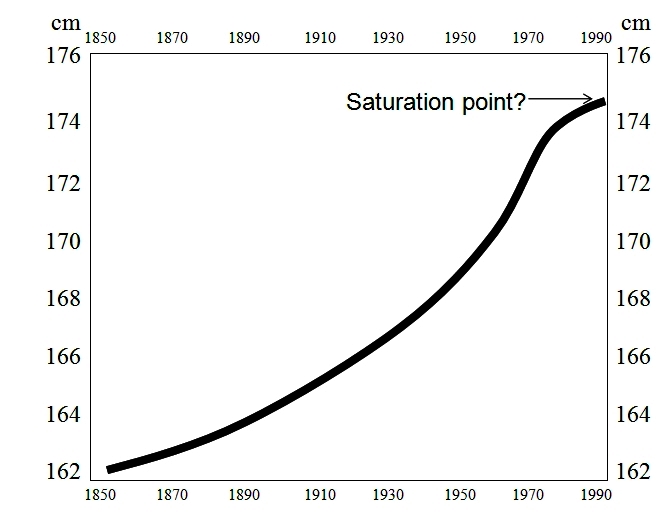
The secular trend in height. Stature at the age of 20 years in Italy from 1854 to 1980, showing the secular trend in height. Height seems to have almost reached a saturation point in 1990. Data from Arcaleni (Econ Hum Biol 2006).^17^

**Figure 6:**
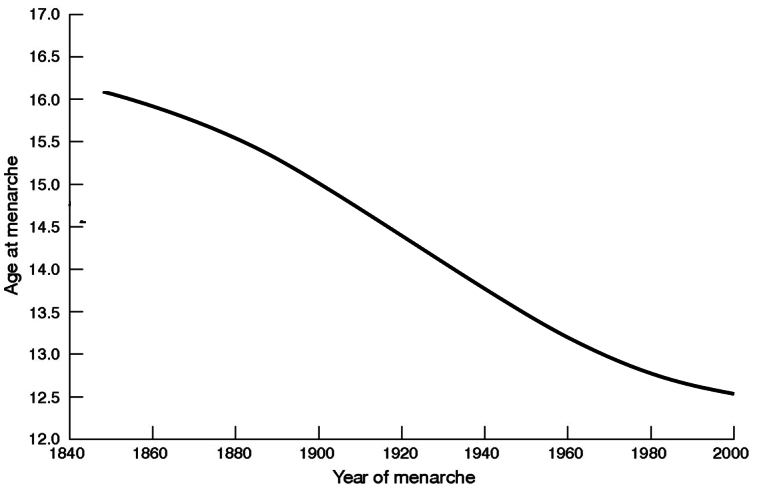
The secular trend in puberty. Declining age of menarche in Europe and the US from 1840 to 2000. Data from Bellis et al., J Epidemiol Community Health (2006).^18^ The lines do not show a saturation point; the trend is expected to continue.

The secular trend in child growth and puberty is a dazzling example of such an adaptation (Figures 5 and 6). Over the six generations of a century and a half between the mid-1850s and 2000s, genes have obviously changed little, and the secular trend exemplifies the degree of available plasticity. If Italian men are now 13 cm taller than they were 150 years ago, and if they indeed reached a saturation point in their height, the plasticity magnitude for height is at least 13 cm. The 4-year difference in menarche between nineteenth- and twentieth-century girls demonstrates the plasticity magnitude for this event with a given genome, and the declining age of menarche does not seem to have reached a saturation point.

Adaptation of height to the environment is also evident in skeletons of ancient Maya that were found in Tikal, Guatemala.^19^ Tomb skeletons (indicating wealth) from the classical period AD 250–400 show an average male length of 172 cm, while the non-tomb (poor) skeletons are 164 cm long. In the late classical period AD 700–900, tomb skeletons are on average 162 cm and non-tomb skeletons 156 cm, showing the impact of poverty on growth of the Maya. Post-conquest (1520) the average length of skeletons is 156 cm – by then everyone was poor.

Such phenotypic plasticity has been described in almost every group of plants and animals, including vertebrates and humans.^20^ In the most extreme case, plastic changes of a trait are able completely to move a population to a new fitness optimum, and no genetic adaptation is required. In cases where a plastic change in the mean is not sufficient to shift a population to a new optimum, it can allow a population to persist until sufficient adaptive genetic changes occur.^21^ Plastic responses during early development have particularly important fitness consequences since they can lead to permanent and profound modifications of morphology or physiology.^22^ In fact, for most trade-offs to function, they must be delineated early in development. The impacts of nutritional cues on phenotypic development and the expression of traits such as obesity, the metabolic syndrome, diabetes mellitus type 2, and reproductive performance have been extensively investigated.

The German-American anthropologist Franz Boas (1858–1942) introduced the evolutionary concept of developmental plasticity early in the 1900s as part of his study of human cranial structure; this was expanded in the 1960 by Lasker.^23^ We now recognize two classes of adaptive responses or plasticity.^24^ The first class is anticipatory or predictive adaptive responses, when the developing organism uses environmental cues to forecast the future environment in order to adjust its phenotypic trajectory accordingly. The second class is immediate adaptive responses, which promote short-term maternal or fetal survival but at a cost of trade-offs later in life (developmental plasticity). The window of developmental plasticity extends from before conception to weaning and involves epigenetic responses to environmental influences that exert their effect in early life. These two adaptive responses do not come without a significant cost. Within the potentially adaptive class of responses, the organism may engage in a trade-off between phenotypic changes in order to ensure short-term survival but at the expense of a long-term advantage. Therefore, trade-offs may manifest themselves with longevity at the cost of reduced survival of the juveniles. Trade-offs arise because energy has to be parcel-lated, and individual members of a species make a virtual cost–benefit analysis in order to determine the true value of an adaptive response. Such is the consequence of embryonic fetal development that occurs in a deprived intrauterine environment as a result of limited transplacental nutrient supply. In response, the fetus protects the development of the heart and brain, but at the expense of other organs, resulting in somatic growth retardation.

Underlying this developmental plasticity is the fundamental premise that the physiology of an individual is driven by the induction of a particular developmental program, which is influenced by the prevailing environment existing during a critical developmental period.^25^ While more likely to survive to birth, such neonates are smaller and have higher rates of morbidity and mortality. Given the long-term trade-off in fitness, intrauterine growth restriction (IUGR) is an example of when the immediate adaptive response to environment is cryptically maladaptive.^26^

As a consequence of life conditions under a changing environment, children may be stunted for short or longer periods, be underweight or overweight, and be at risk for disease. In the endocrine jargon, this has been labeled “developmental programming”. The evolutionary language for the same is a “predictive adaptive response”.^27^ Even under good conditions, the stages of human life-history are replete with trade-offs for survival, productivity, and reproduction. Under adverse conditions, trade-offs result in reduced survival, poor growth, constraints on physical activity, and poor reproductive outcomes. Whereas programming implies permanent maladaptive effects that place people at risk for disease, or a pathology in the medical jargon, predictive adaptation considers the phenotypic changes as constructively adaptive, and at two levels: 1) short-term adaptive responses for immediate survival, and 2) predictive responses required to ensure postnatal survival to reproductive age to increase reproductive fitness. To adapt to a changing environment, an organism has to be able to modify stably the expression of its genome. It is the changeability that is genetically determined and the changes that manifest the phenotypic plasticity. Organismal size, including child growth, is probably the supreme case in point of this paradigm.

The initial^28^ and then plethora of reports on the consequences of the 1944–1945 Dutch famine illustrate these concepts at their extreme. Exposure to a relatively short famine of 6 months early in life had lifelong effects on health, and these effects varied depending on the timing and length of exposure and its recovery timing and period. Exposure to famine during gestation resulted in later impaired glucose tolerance, obesity, coronary heart disease, hyperlipidemia, and hypertension, but also in antisocial personality, schizophrenia, and affective disorders.^29^ Exposure to famine during childhood resulted in changes in reproductive function, earlier menopause, and changes in insulin-like growth factor-1 levels.

Following the initial description by Barker et al.,^30^ another plethora of reports described the health consequences of intrauterine growth retardation. Intrauterine programming became the doctrine that explained these phenomena: the ultimate outcome is an evidence of pathology. Well nourished mothers have offspring who are adapted to affluent conditions, whereas mothers on a low level of nutrition have offspring who are adapted to lean environments.^31^ If the mother’s “forecast” of her offspring’s future environment is incorrect, the health of her offspring in a mismatched environment may suffer severely.^32^ Understanding the evolutionary background sets the developmental origins of ill health in humans in context and has profound implications for public health.^31^

Kuzawa proposed that an organism has a metabolic potential in excess of survival requirements that he called productivity, supporting growth before being shunted into reproduction after growth ceases.^33^ In his “intergenerational phenotypic inertia” model, he predicted that plasticity in growth rate will be positively correlated with components of future adult reproductive expenditure. Thus, early nutrition or growth rate predicts offspring size in females, and increased somatic investment related to reproductive strategy in males. Indeed, population birth-weight and sexual size dimorphism are predicted to increase in response to improvements in early nutrition.^33^ This is perpetuated not merely during the life cycle but across generations: in females, growth rate predicts future nutritional investment in reproduction, which in turn determines fetal growth rate in the next generation. Kuzawa suggested that growth and reproduction serve as mutually defining templates, thus creating a phenotypic bridge, allowing ecologic information to be maintained during ontogeny and transmitted to offspring.^33^ Resetting of metabolic production in response to maternal nutritional cues may serve a broader goal of integrating trans-generational nutritional for adjusting long-term strategy.

Ernst Mayr (1904–2005) referred to phenotypic accommodation to the environment as *soft inheritance* (Lamarckism), in contrast with *solid inheritance* by gene structure. However, solid evidence is now available for molecular mechanisms of epigenetics that underlie adaptive plasticity.

## INFANCY–CHILDHOOD TRANSITION: DETERMINATION OF ADULT STATURE

There is an evolutionary adaptive strategy of plasticity in the timing of the transition from infancy into childhood in order to match environmental cues and energy supply.^34^ We proposed that humans evolved to withstand energy crises by decreasing their body size, and that evolutionary short-term adaptations to energy crises utilize probably epigenetic mechanisms that modify the transition into childhood, culminating in short stature.

Pediatricians speak of an infant’s failure to thrive (FTT) when his weight or height gains are insufficient. This is observed mostly during the childhood onset period of 6–12 months and beyond. It has been shown that whether the cause was organic or non-organic, such children often remain short,^35^,^36^ and the mechanism of that delay is a delay in the infancy–childhood transition (DICT).^34^

Based on the predictive adaptive response theory, the expected response to a secure environment includes the investment in large body size, whereas the expected response to a threatening environment will include a reduction in body size. At the same time, predictive responses to resist a threatening and difficult environment may include altered hypothalamic–pituitary–adrenal axis, altered behavior and anxiety, increased appetite and tendency to store fat, altered food preference, reduced motor behavior and skeletal muscle mass and strength, altered vasculature function, altered insulin release and action, and leptin resistance.^37^

**Figure 7:**
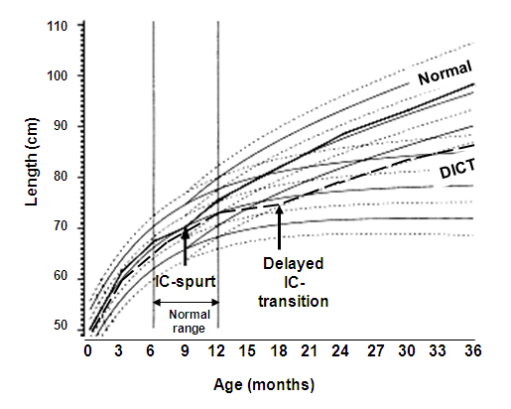
The Infancy–childhood–puberty (ICP) model; mean and ± 1, 2, and 3 SDS lines. The childhood growth stage sets on in Sweden between 6 and 12 months of age, and when delayed (delayed infancy childhood transition, DICT) it has a permanent effect on final adult height. Adapted with permission from Hochberg Z et al., Pediatr Res (2008).^34^

Based on analysis of growth parameters, the infancy, childhood, and puberty (ICP) growth model of Johan Karlberg divided human growth into three successive and partly superimposed stages that reflect the endocrine control mech-anisms of the growth process^38^,^39^ (Figure 7). While this model ignores juvenility, it is still valid in the argumentation for the infancy–childhood growth transition. The infancy stage of the ICP model has been assumed to begin at mid-gestation and to tail off at approximately 2–3 years of age, representing the postnatal extension of fetal growth, and is regarded as being nutrition-dependent and closely linked to the action of insulin-like growth factors (IGFs). The childhood growth stage starts in affluent Western countries between 6 and 12 months of age^34^,^40^,^41^ and continues through puberty until growth ceases at attainment of adulthood. Thus, the ICP model proposes a period of transition, whereby the initiation of the childhood growth stage overlaps with the infancy growth stage and the infantile life-history stage, as defined by weaning in traditional societies at 2–3 years of age.

In affluent Sweden, the childhood growth stage begins between 6 and 12 months of age (mean ± 2 SD), with a mean age of 9 months.^40^,^41^ The mean age for transition is 10 months in Israel, 11 months in Shanghai,^42^ 13 months in Pakistan, and 30 months in Malawi. Whereas about 3% of Swedish infants have an infancy–childhood transition beyond the age of 12 months, 7% of Israeli infants show such a delay, and about 11% of the infants in Shanghai fall into that category.

Children with DICT have both normal infantile and normal childhood growth rates, indicating normally functioning growth control mechanisms during both of these life-history stages (Figure 7).^34^ Their only abnormal experience is the late transition age. As a result, children with DICT are longer at birth and during infancy than short children with normal transition age, whereas their heights during later life-history stages are comparable. DICT has been reported in as many as 50% of children with “idiopathic” (reason unknown) short stature.^34^,^43^ This effect is time-dependent, with a longer delay resulting in shorter pre-pubertal stature^44^ and final adult height.^34^

## CONCLUSIONS

Hereditary, environmental, and stochastic factors determine a child’s growth in his unique environment, but their relative contributions to the phenotypic outcome and the extent of stochastic programming that is required to alter human phenotypes are not known because few data are available.^24^ If the environment can influence growth and developmental trajectories during pre-adult life-history stages and later life outcomes, how do epigenetic events influence the transition from one life-history stage to the next, growth and puberty at the molecular level?

Growth and puberty are regulated by insulin, growth hormone, the IGFs, and the sex hormones, to mention a few of the control hormones. These hormones drive the rate of growth and development, but it is unclear how the environment determines the timing of the different phases of developmental events and the quantity of growth.

Epigenetic mechanisms potentially play an important role in our life-history and the transitions. Environmental influences during embryonic and early life development can permanently alter epigenetic gene regulation, which in turn can result in imprinting and reprogramming of the epigenome and influence child growth, maturation, development, and body composition in later life-history stages. The mechanisms by which cues about nutrient availability in the uterus and postnatal environment are transmitted to the offspring and by which different stable phenotypes are induced are still unknown.

Since no other animal has a similar pre-adult life-history to that of humans, an obvious question is whether the findings from any experimental animal can be extrapolated to humans. Focusing specifically on the needs and opportunities in child health, we need better phenotypic assessments than currently in order to define study populations.

Perhaps the most fundamental set of questions raised by a life-history approach to child growth concerns how the unique growth pattern of each child in his given genetic background and current environment best serves his reproductive fitness. Further research is required to understand the mechanisms underlying the energy allocation process toward growth during pre-adult stages. Another direction will be to understand how these mechanisms interact with socio-economic conditions in generating behaviors that affect life-history and growth. Finally, the obesity epidemics that have affected us in recent decades require special attention for evolution under unlimited resources.

## Abbreviations:

DICT: delayed infancy–childhood transition;
IGF: insulin-like growth factor;
IUGR: intrauterine growth restriction;
kya: thousand years ago;
mya: million years ago.
